# Genetic and bioinformatic analyses of the expression and function of PI3K regulatory subunit PIK3R3 in an Asian patient gastric cancer library

**DOI:** 10.1186/1755-8794-5-34

**Published:** 2012-08-09

**Authors:** Jin Zhou, Geng Bo Chen, Yew Chung Tang, Rohit Anthony Sinha, Yonghui Wu, Chui Sun Yap, Guihua Wang, Junbo Hu, Xianmin Xia, Patrick Tan, Liang Kee Goh, Paul Michael Yen

**Affiliations:** 1 Laboratory of Hormonal Regulation, Cardiovascular and Metabolic Disorders Program, Duke-National University of Singapore Graduate Medical School, Singapore; 2 Laboratory of Genomic Oncology, Cancer and Stem Cell Program, Duke-National University of Singapore Graduate Medical School, Singapore; 3 Laboratory of Computational Biology, Cancer and Stem Cell Program, Duke-National University of Singapore Graduate Medical School, Singapore; 4 Laboratory of Computational Systems Biology and Human Genetics, Neuroscience and Behavioral Disorder Program, Duke-National University of Singapore Graduate Medical School, Singapore; 5 Cancer Research Institute, Tongji Hospital, Huazhong University of Science and Technology, Wuhan, 430030, China; 6 Department of Medical Oncology, National Cancer Centre, Singapore; 7 Saw Swee Hock School of Public Health, National University of Singapore, Singapore; 8 Cellular and Molecular Research, National Cancer Centre, Singapore; 9 Cancer Science Institute of Singapore, National University of Singapore, Singapore; 10 Genome Institute of Singapore, Singapore

## Abstract

**Background:**

While there is strong evidence for phosphatidylinositol 3-kinase (PI3K) involvement in cancer development, there is limited information about the role of PI3K regulatory subunits. PIK3R3, the gene that encodes the PI3K regulatory subunit p55γ, is over-expressed in glioblastoma and ovarian cancers, but its expression in gastric cancer (GC) is not known. We thus used genetic and bioinformatic approaches to examine PIK3R3 expression and function in GC, the second leading cause of cancer mortality world-wide and highly prevalent among Asians.

**Methods:**

Primary GC and matched non-neoplastic mucosa tissue specimens from a unique Asian patient gastric cancer library were comprehensively profiled with platforms that measured genome-wide mRNA expression, DNA copy number variation, and DNA methylation status. Function of PIK3R3 was predicted by IPA pathway analysis of co-regulated genes with PIK3R3, and further investigated by siRNA knockdown studies. Cell proliferation was estimated by crystal violet dye elution and BrdU incorporation assay. Cell cycle distribution was analysed by FACS.

**Results:**

PIK3R3 was significantly up-regulated in GC specimens (n = 126, p < 0.05), and 9.5 to 15% tumors showed more than 2 fold increase compare to the paired mucosa tissues. IPA pathway analysis showed that PIK3R3 promoted cellular growth and proliferation. Knockdown of PIK3R3 decreased the growth of GC cells, induced G0/G1 cell cycle arrest, decreased retinoblastoma protein (Rb) phosphorylation, cyclin D1, and PCNA expression.

**Conclusion:**

Using a combination of genetic, bioinformatic, and molecular biological approaches, we showed that PIK3R3 was up-regulated in GC and promoted cell cycle progression and proliferation; and thus may be a potential new therapeutic target for GC.

## Background

Class IA Phosphatidylinositol 3’-kinases (PI3K) is a heterodimer that consists of a p110 catalytic subunit and a p85 regulatory subunit. The catalytic subunit isoforms p110α, p110β and p110δ are encoded by three genes — *PIK3CA*, *PIK3CB* and *PIK3CD,* respectively [1]. The role of catalytic subunits in a wide range of cellular processes associated with cancer development and progression is well established [2]. The PIK3CA gene is one of the best studied oncogenes, and is amplified, overexpressed, or frequently mutated in many cancers, including GC [2,3]. PIK3CB is the principal isoform involved in mediating PTEN-deficient tumourigenesis [4]. In addition, PIK3CD has also emerged as a key therapeutic target for haematological malignancies [5], notably acute myeloid leukaemia (AML). Consequently, targeting catalytic subunits represents an important strategy for the development of novel cancer therapeutics.

In contrast, current understanding of the role of regulatory subunits in tumorigenesis has been limited. Three genes, PIK3R1, PIK3R2 and PIK3R3, encode the p85α, p85β and p55γ isoforms of the p85 regulatory subunit, respectively [6]. PIK3R1, the inhibitory subunit of PI3K, is mutated in primary colorectal and endometrial cancer tumors [7,8], and ectopic expression of some of those mutations increased the pAKT level in U2OS cells. Interestingly, PIK3R3 has increased expression in glioblastoma multiforme and ovarian cancer [9,10]. Knockdown of PIK3R3 inhibits IGF2-induced cell growth in glioblastoma multiforme [9] and induces apoptosis in ovarian cancer cells [10]. Taken together, these results suggest an oncogenic role for PIK3R3 in these cancers.

GC is the second leading cause of global cancer mortality and is highly prevalent among Asians [11]. Most GC patients are diagnosed with late stage disease and the overall 5-year survival rate is <24% [12-14]. Deregulation of canonical oncogenic pathways such as E2F, K-RAS, p53, and Wnt/b-catenin signaling are known to occur with varying frequencies in GC [15-17], suggesting that GC is a heterogeneous disease with multiple molecular defects. Although PIK3R3 is overexpressed in several cancers, little is known about the expression and functional role of PIK3R3 in GC. To address these issues, we used genetic and bioinformatic approaches to interrogate a unique library of 126 paired GC samples and matched non-neoplastic mucosa tissues from Asian GC patients.

## Methods

### Human cancer specimens and cell lines

We created a library of 126 primary gastric tumors and their matched non-neoplastic mucosa tissues from 126 Asian patients that were originally stored at the SingHealth Tissue Repository, an institutional resource of National Cancer Centre of Singapore and Singapore General Hospital. All patient samples were obtained with informed patient consent and approvals from Institutional Review Boards and Ethics Committees. GC cell lines HGC-27, KATO III, AGS cells were purchased from the American Type Culture Collection. MKN7, TMK1 and IM95 cells were obtained from the Japan Health Science Research Resource Bank. Cell lines were maintained in a humidified atmosphere containing 5% CO2 at 37°C. HGC-27, IM95 cells were cultured in DMEM medium supplemented with 10% FBS (Sigma); AGS, TMK1 and KATO III and MKN7 were cultured in RPMI 1640 medium supplemented with 10% FBS.

### Microarray Profiling & Pre-processing

Genomic DNA was extracted from flash-frozen tissues using a Qiagen genomic DNA extraction kit. Total RNAs was extracted using Trizol (Invitrogen, CA), digested with RNase free DNase (RQ1 DNase, Promega), and subsequently purified using an RNeasy Mini kit (Qiagen,CA). Genome-wide mRNA expression was profiled using Affymetrix GeneChip Human Genome U133 Plus 2.0 array. Copy number analysis was profiled using Affymetrix Human SNP array 6.0. DNA methylation analysis was profiled using Illumina Infinium methylation assay. Profiling on the individual microarray platforms was done according to manufacturer’s specifications.

The raw microarray data was pre-processed using the respective platform software from the manufacturer: mRNA and SNP data using Affymetrix’s Genome Studio and Genotyping Console respectively, and DNA methylation using Illumina’s BeadStudio. For SNP data, the normal gastric samples were used as the reference for normalization. The copy number segmentation was generated using Circular Binary Segmentation (CBS) from the R package DNA copy [18] using default settings. Gene-based copy number alterations were obtained by averaging the segments within each gene. Amplification and deletion were called using the threshold of 0.3 and −0.3 respectively [19].

The microarray data has been deposited into the National Centre for Biotechnology Information’s (NCBI) Gene Expression Omnibus (GEO) website, series accession number GSE31168 (SNP6) and GSE15460 (mRNA).

### PIK3R3 co-regulated gene and IPA pathway analysis

Co-regulated genes with PIK3R3 were elucidated using Pearson correlation on the mRNA data. We focused on probes that were specifically targeting the genes (*i.e.,* probes with suffix ‘_at’ and ‘_a_at’) and significant correlation after correcting for multiple testing (p < 1e-5). Pathways involving the probes were assessed using Ingenuity Pathway Analysis software ( http://www.ingenuity.com). All statistical analyses were computed using the R statistical package.

### RNA Extraction and RT-PCR

Total cellular RNA was isolated with the High Pure RNA isolation Kit (Roche) following the manufacturer's protocol and total RNA was quantified with a Nanodrop ND-1000 spectrophotometer. Total RNA (1 μg) was reverse-transcribed using iSCRIPT cDNA synthesis kit (Bio-rad) under condtions defined by the supplier. cDNA was quantified by real-time PCR on the Rotor-Gene Q System (Qiagen). PIK3R3 forward primer: GAGAGGGGAATGAAAAGGAGA, and reverse primer: ATCATGAATCTCACCCAGACG [10]. β-actin forward primer: AGAGCCTCGCCTTTGCCGAT, and reverse primer: TTGCACATGCCGGAGCCGTT; GAPDH forward primer: TCTTTTGCGTCGCCAGCCGA, and reverse primer: CCAGGCGCCCAATACGACCA. PCR was done using QuantiFast SYBR Green PCR Kit (Qiagen) according to manufacturer’s instructions.

To test the effect of LY294002 on PIK3R3 expression, the complete medium was replaced by serum free medium and LY294002 (20 μM/L, Sigma) was added. The total RNA was extracted 24 h after LY294002 treatment.

### PIK3R3 siRNA transfection

Two PIK3R3 siRNAs ordered from Santa Cruz (sc-39124) or Invitrogen (s16152) were used to knock down PIK3R3 in GC cells. HGC-27 or TMK1 cells were seeded into 12 or 6-well plates in complete medium and cultured overnight. Then the medium was replaced with opti-MEM medium (Invitrogen) containing PIK3R3 or control siRNA and Lipofectamine RNAimax (Invitrogen) according to the manufacturer’s recommendations. 48 h after transfection, cell lysates were prepared for Western blot analysis for detection of PIK3R3 expression.

### Cell proliferation assay

Cell proliferation assay was done as previously described [20,21]. Briefly, 24 h after siRNA transfection, the HGC27 cells were trypsinized, resuspended in 1.1 ml complete culture medium, and re-seeded into 4 new 12-well plates at 250 μl per well. The cells were fixed 1 to 4 days after re-seeding, respectively, and stained with crystal violet. Using 10% acetic acid, dye was extracted, and absorbance at 595 nm was measured using a multiwall spectrophotometer (Bio-Rad).

### Measurement of BrdU incorporation

48 h after siRNA transfection, HGC27 cells cultured in 6 well-plates were incubated with BrdU (10 μg/ml) for 7 min. The cells were detached, fixed and stained using FITC BrdU Flow Kit (BD Pharmingen) following the manufacturer's protocol. DNA synthesis was determined by flow cytometer.

### Annexin-V/propidium iodide staining

Annexin V staining was detected by flow cytometry using the FITC Annexin V Apoptosis Detection Kit I (BD Pharmingen). 48 h after siRNA transfection, Both floating and adherent HGC27 cells were collected. Then the cells were washed and stained following the manufacturer's protocol. Apoptotic cells were determined by flow cytometer.

### Cell cycle analysis

48 h after siRNA transfection, HGC-27 cells were harvested, washed twice with cold phosphate buffered saline (PBS, pH 7.4), and fixed with 70% ethanol/30% PBS at 4°C overnight. The fixed cells were incubated with 0.5 ml PBS containing 10 μg/ml RNase for 30 min at 37°C, then stained with 20 μg/ml propidium iodide (PI, Sigma) for 30 min in the dark at room temperature, and finally analyzed on a FACS cytometer (Beckman FC5000). A minimum of 1 × 10^4^ cells/sample was evaluated.

### Western blot analysis

Cells were harvested, and lysed 48 h after siRNA transfection. Proteins were separated by SDS–PAGE under reducing conditions and transferred to nitrocellulose membranes. Membranes were blocked with 5% nonfat milk in phosphate-buffered saline with 0.1% tween 20 (PBST), and the membranes for PI3KR3 blot were blocked with 2% gelatine, 0.2% FBS in PBST [22]. The blots were incubated overnight at 4°C with the following antibodies: anti-PIK3R3 antibody, anti-beta actin antibody (Santa Cruz), anti-phospho-RB (Ser 780), anti-cyclin D1, anti- phospho-p38 MAPK antibody (Thr180/Tyr 182), anti-phospho-AKT (Ser 473), anti-phospho-AKT (Ser 308), anti-phospho-m-TOR (Ser 2448) (Cell signaling Technology), anti-PCNA antibody (Millipore). Immunoblot analysis was performed using an enhanced chemiluminescence procedure (GE Healthcare).

## Results

### PIK3R3 mRNA is over-expressed in a subgroup of GC

**Figure 1:**
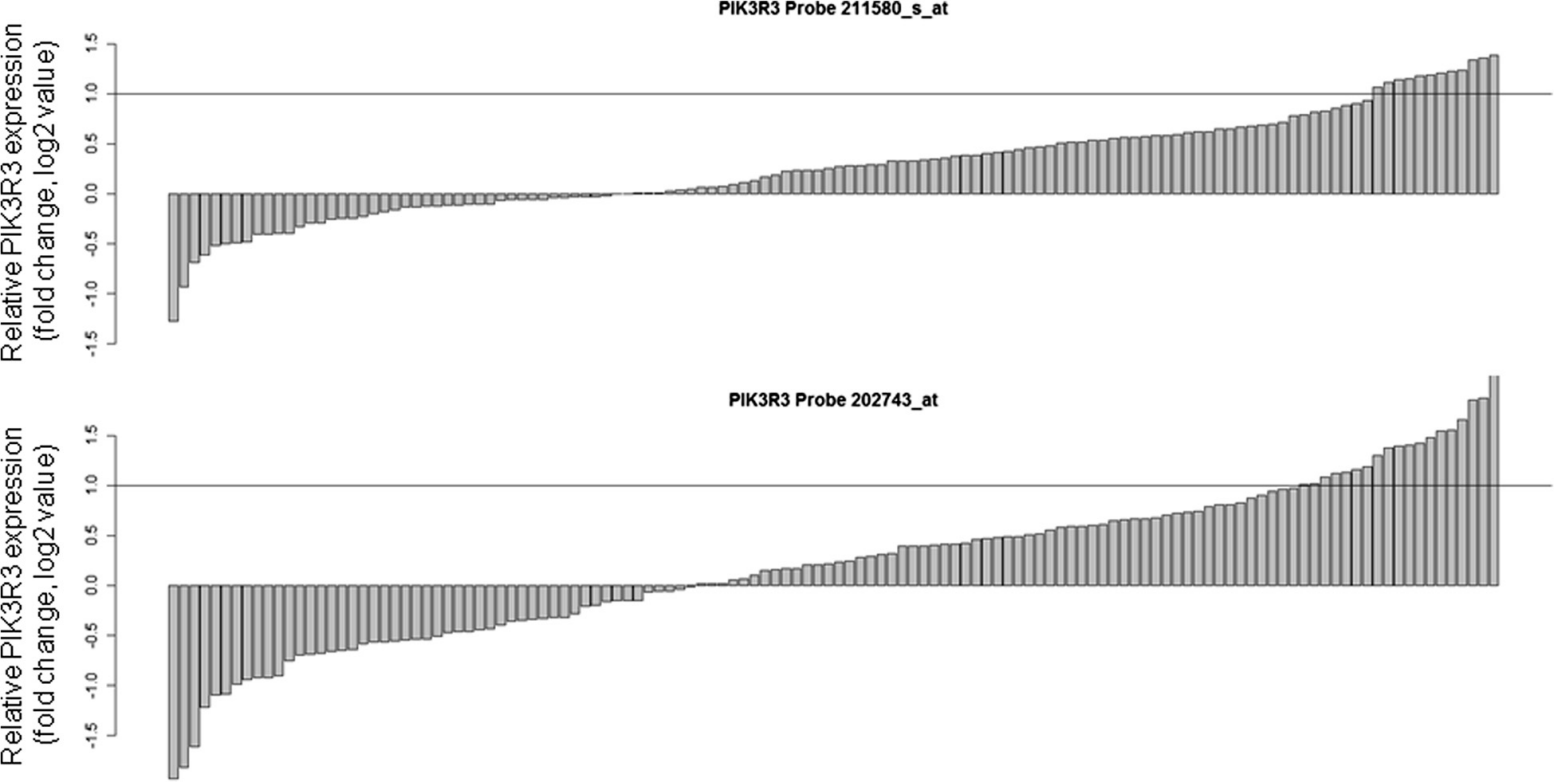
** PIK3R3 mRNA expression in 126 GC and paired non-neoplastic mucosa.(A)** The fold change (log 2 value) of PIK3R3 mRNA expression between the GC and their paired non-neoplastic mucosa. 12 (211580_s) or 19 (202743_at) cases showed more than 2 fold increase of PIK3R3.

We examined the PIK3R3 mRNA expression of 126 gastric adenocarcinoma samples and matched non-neoplastic mucosa control gastric tissues, profiled on Affymetrix GeneChip Human Genome U133 Plus 2.0 arrays. We identified cancers that expressed elevated PIK3R3 by comparing PIK3R3 mRNA expression in the tumor samples with their expression in control tissues from the same patients. Two probe sets (202743_at and 211580_s_at) interrogated PIK3R3 mRNA levels in these arrays, and thus provided two independent read-outs of PIK3R3 gene expression. PIK3R3 was significantly up-regulated in cancer specimens (p < 0.05) and considered over-expressed if the probe sets measured a greater than 2-fold increase in PIK3R3 expression in the tumor sample compared to its control. Thus, 19 (15%) or 12 (9.5%) samples exhibited PIK3R3 over-expression according to different probe sets (Figure 1).

### Knockdown of PIK3R3 expression decreases cell proliferation in GC cells

**Figure 2:**
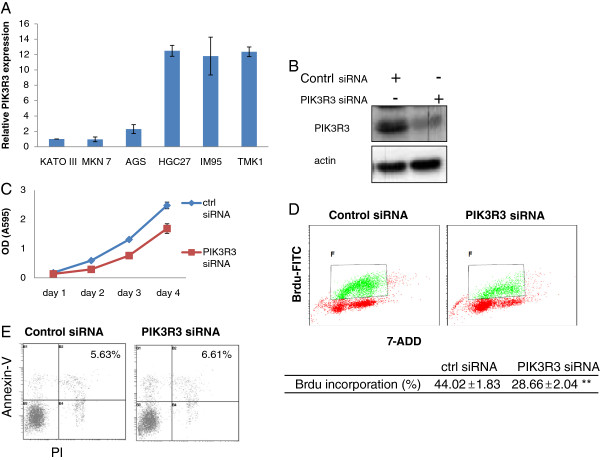
** Knock down PIK3R3 by siRNA (sc-39124, Santa Cruz) inhibited the growth of HGC27 GC cells.(A)** PIK3R3 mRNA level in 4 GC lines measured by quantitative RT-PCR, and the relative PIK3R3 mRNA level was calculated by comparison against KATO III. Shown are the mean +/− s.d. of triplicate samples. **(B)** Western blot of PIK3R3 knockdown in transfected cells. **(C)** Cell number (measured by optical density (O.D.)) after knockdown by PIK3R3 siRNA. **(D)** DNA synthesis (BrdU incorporation) after knockdown of PIK3R3. The data in these experiments are expressed as means ± S.D. from three independent experiments. The significance was assessed by paired student’s *t*-test. *P < 0.05 comparing knockdown with control. **(E)** Cell death measured by Annexin-V/PI staining.

In order to understand the potential role of PIK3R3 in GC, pathway analyses (Ingenuity IPA software) was performed on genes co-regulated with PIK3R3. Pathway analysis results highly suggested that PIK3R3 was involved in cell growth and proliferation (Additional file 1: Figure S1). To further investigate the potential effect of PIK3R3 expression on cell proliferation, we first tested the expression of PIK3R3 in 6 GC cell lines (Figure 2A). HGC27, TMK1 and IM95 had significantly higher PIK3R3 expression than KATO III, AGS and MNK7. We next used PIK3R3 siRNA to specifically knock down PIK3R3 protein expression in HGC27 GC cells, and confirmed the knockdown efficiency by Western blotting (Figure 2B). Knockdown of PIK3R3 significantly decreased cell proliferation based upon both crystal violet dye elution and BrdU incorporation assay (Figures 2C and D). Additionally, PIK3R3 knockdown did not increase in the early apoptotic cell populations (Figure 2E). The results were confirmed in HGC27 cells using another PIK3R3 siRNA (Additional file 2: Figure S2).

### Knockdown of PIK3R3 causes G0/G1 cell cycle arrest

**Figure 3:**
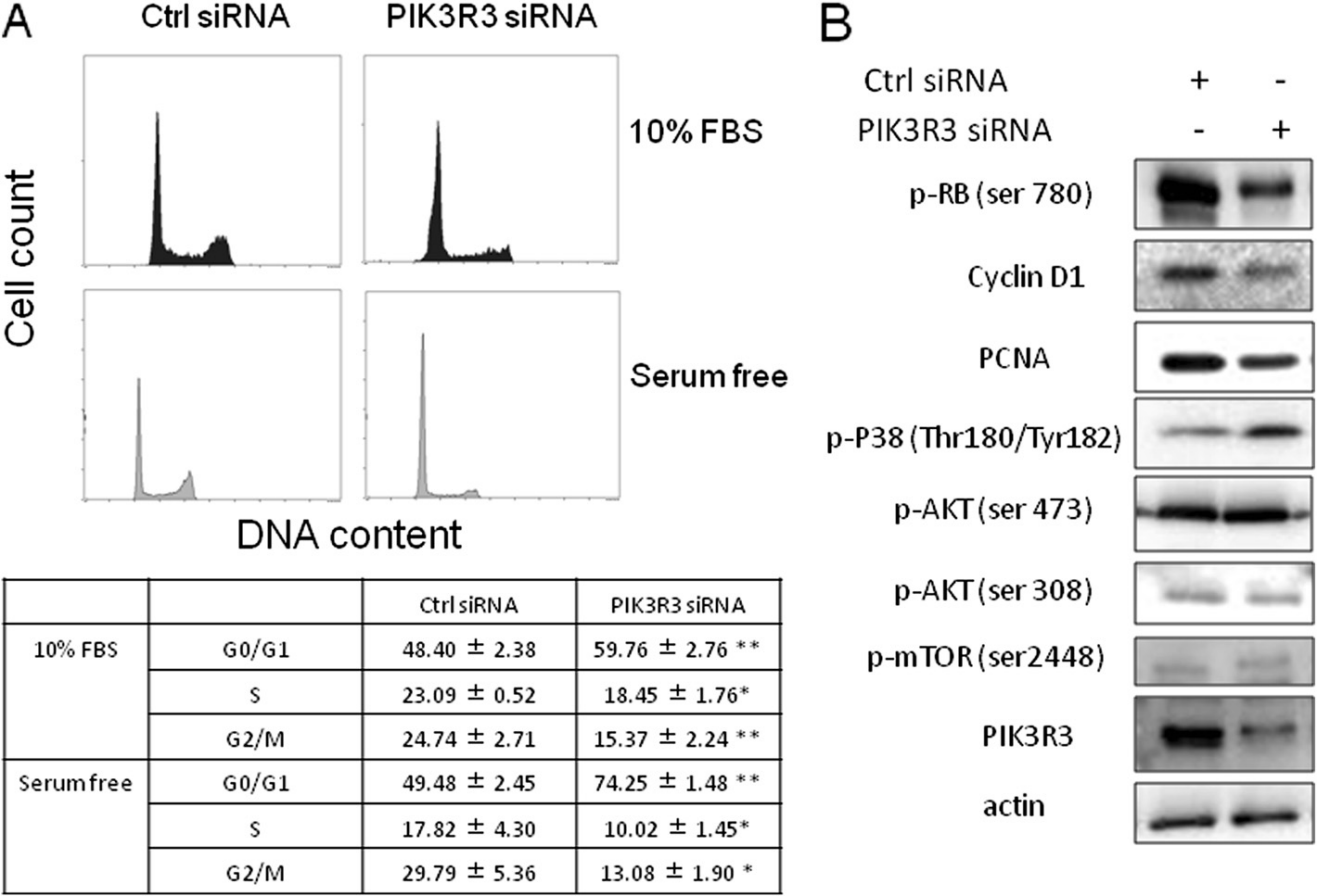
** Effects of PIK3R3 knockdown on cell cycle progression and cell signaling of HGC27 GC cells.(A)** The effect of PIK3R3 knockdown on cell cycle progression in HGC27 cells. The cells were cultured in complete medium for 48 h or 24 h followed by another 24 h in serum free medium after siRNA transfection. The cells then were harvested for cell cycle analysis. The data are expressed as means ± S.D. from three independent experiments. The significance was assessed by paired student’s *t*-test. *P < 0.05 and **P < 0.01 compared with control. **(B)** Western blot analysis of the effects of PIK3R3 knockdown on cell signaling. Total cell extracts were probed with antibodies to cyclin D1, phospho-Rb (p-Rb), PCNA, phospho-AKT (p-AKT), phospho-mTOR (p-mTOR), PIK3R3, actin (loading control) as indicated.

Since PIK3R3 knockdown decreased cell proliferation, we examined whether PIK3R3 knockdown also caused cell cycle arrest at the G0/G1 phase by PI staining and FACS analyses (Figure 3A). We observed that PIK3R3 knockdown increased cells in G_0_/G_1_ arrest (59.8% *vs.* 48.4%). Furthermore, the percentage of knockdown cells in G_0_/G_1_ cell cycle arrest increased when cells were deprived of growth factors in culture media conditions (Figure 3A). We also found increased G0/G1 cell cycle arrest in TMK1 GC cells (Additional file 3: Figure S3A). Taken together, our results suggest that the main effect of PIK3R3 knockdown is inhibition of cell proliferation by G0/G1 cell cycle arrest rather than induction of cell apoptosis.

### Knockdown of PIK3R3 decreases Rb phosphorylation and cyclin D1 protein expression but does not affect basal pAkt level

To understand better the functional role of PIK3R3 on cell proliferation, we examined the expression of various molecular markers in the PIK3R3 knockdown cells. We found reduced cyclin D1 levels and decreased phosphorylation of Rb protein, both of which are associated with G0/G1 arrest (Figure 3B and Additional file 3: Figure S3B). Expression of the cell proliferation marker, PCNA, also was reduced (Figure 3B). Interestingly, phosphorylation of Akt and mTOR was unaffected by PIK3R3 knockdown (Figure 3B and Additional file 3: Figure S3), suggesting that over-expression of PIK3R3 does not primarily drive cell proliferation through PI3K-pAkt signaling.

### PIK3R3 over-expression can be regulated by PIK3CA

**Figure 4:**
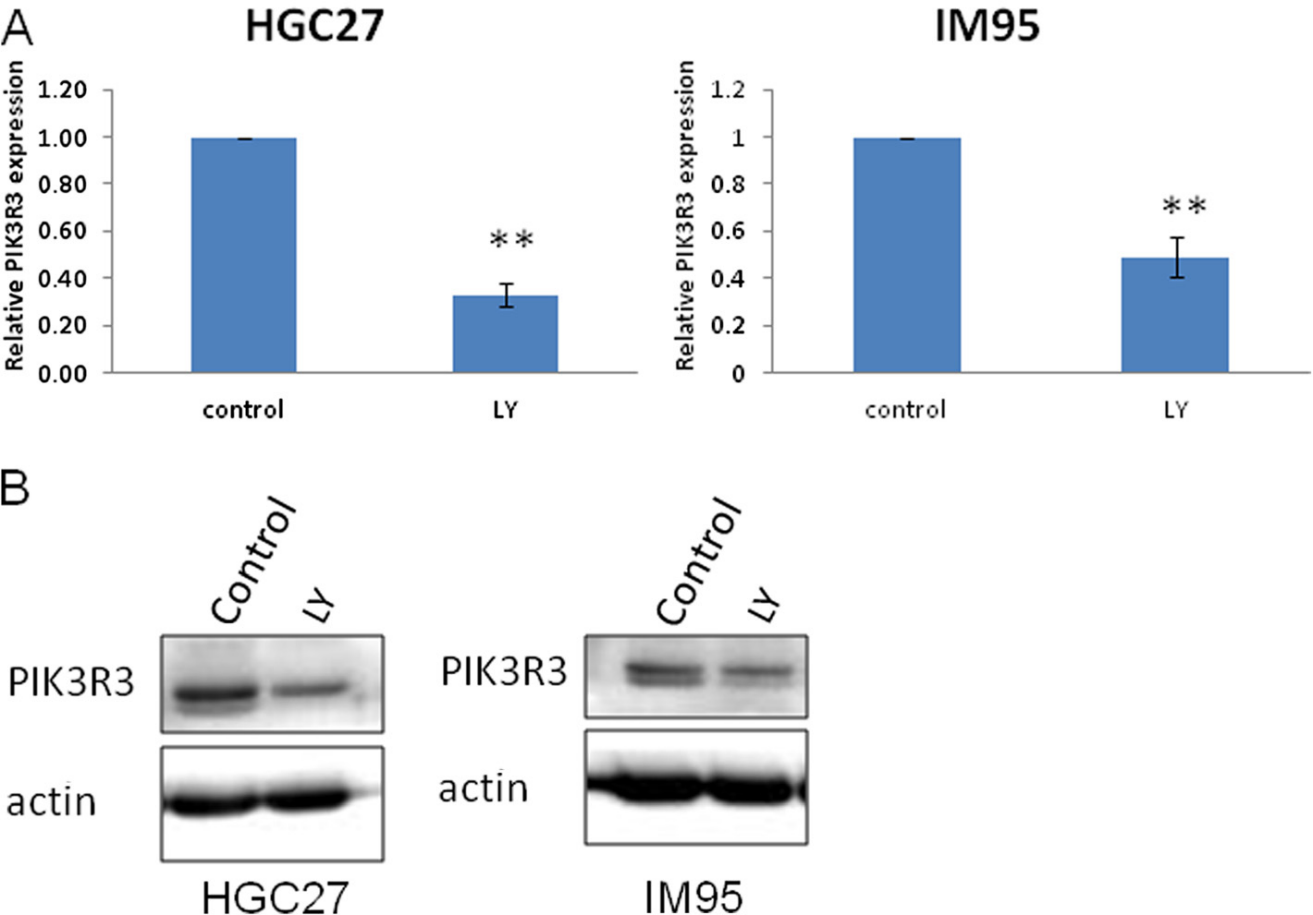
** Effects of inhibition of PIK3CA signaling on the expression of PIK3R3.** HGC27 and IM95 GC cells were seeded in complete medium overnight. The complete medium was replaced by serum free medium, and PIK3CA inhibitor LY294002 was added. Total RNA or cell lysates were harvested 24 h after treatment. The relative PIK3R3 mRNA level **(A)** or protein level **(B)** was measured by quantitative RT-PCR or western blot.

47 of the 126 paired GC and control tissues were comprehensively profiled on multi-omics platforms that measured genome-wide mRNA expression, DNA copy number variation, and DNA methylation status. This multi-platform allowed us to investigate whether structural genomic changes or epigenetic regulation accounted for the up-regulation of PIK3R3. However, we did not observe any PIK3R3 gene amplification (*i.e.* average segments within the gene were < 0.3, Additional file 4: Figure S4A), or CpG island de-methylation in the GC with high PIK3R3 mRNA expression (Additional file 4: Figure S4B). Additionally, there was no consistent significant correlation between either PIK3R3 copy number and mRNA level, or CpG island methylation and the mRNA expression level (Additional file 4: Figure S4). Taken together, these results indicate that some other mechanism accounted for the increased PIK3R3 mRNA expression in the high-expressing GC. Recently, several studies have suggested that activated PI3K signaling induces the expression of PIK3R3 [23,24]. We thus investigated this possibility by assessing the correlation of mRNA expression of PIK3R3 and PIK3CA, the p110 α catalytic subunit of PI3K. We found that PIK3CA and PIK3R3 showed a significant positive correlation in 126 GC (Additional file 5: Figure S5). Furthermore, PIK3CA inhibitor LY294002 treatment decreased PIK3R3 mRNA and protein levels in HGC27 and IM95 cells (Figure 4). 

## Discussion

Although the roles of the PI3K catalytic subunit in tumorigenesis is well established, current understanding of the function of PI3K regulatory subunits such as PIK3R3 is still limited. A previous study showed that PIK3R3 gene had copy number gain in high-grade glioma, and the Comparative Genomic Hybridization (CGH) ratios for this locus were positively-correlated with proliferation signatures [25]. Furthermore, the IGF2-PIK3R3 signaling axis was involved in promoting the growth of a subclass of highly aggressive human glioblastomas [9]. In this study, we compared PIK3R3 expression in 126 paired GC with non-neoplastic gastric mucosa control tissues from the same patients, and found that approximately 9.5% to 15% of GC showed increased PIK3R3 expression. IPA pathway analysis suggested a key role of PIK3R3 in cell proliferation. Knockdown of PIK3R3 by siRNA decreased HGC27 GC cell proliferation and induced cell cycle blockade at G_0_/G_1_. Taken together, these results suggest that PIK3R3 stimulates cell proliferation in GC and may be a potential therapeutic target for a subgroup of GC.

PIK3R3 shares significant sequence identity with PIK3R1 and PIK3R2 regulatory subunit in a proline-rich motif and two Src homology 2 (SH2) domains; however, it has an unique 24-residue NH2 terminus [26]. PIK3R3 binds to Rb via this unique region as the N-terminal 24 amino acids of PIK3R3 (N24) are sufficient for binding to Rb [27,28]. Ectopic expression of N24 also inhibited cyclin D1 and E promoter activity and caused G_0_/G_1_ phase arrest in several cancer cell lines [27]. Likewise, in the current study, knockdown of PIK3R3 caused G_0_/G_1_ cell cycle arrest in HGC27 and TMK1 GC cells. Consistent with this observation, Rb phosphorylation, cyclin D1, and PCNA protein levels also were decreased after PIK3R3 knockdown. Interestingly, the pAkt levels did not change after PIK3R3 knockdown. Additionally, when PIK3R3 was overexpressed in AGS cells, a low PIK3R3-expressing gastric cancer cell line, we did not observe any significant change in phosphorylation of Akt (data not shown). Taken together, these findings show that both increased and decreased expression of PIK3R3 do not affect pAkt levels in these cell lines under our experimental conditions. Of note, we have observed similar effects in several colon cancer cell lines and MCF7 breast cancer cells ( [29]and unpublished data). This lack of change in pAkt levels may be due to either preferential nuclear localization of PIK3R3 [27] or low PIK3R3 expression relative to p85 regulatory subunit isoforms. In contrast to our findings, Soroceau *et al.* found decreased pAkt in G96 glioblastoma cells after PIK3R3 knockdown which may be due to different experimental conditions or different tissue origins of cell lines as PIK3R3 expression is high in the brain [9]. Nevertheless, our current and previous studies suggest that PIK3R3 can promote cell growth through a novel signaling pathway by regulating Rb phosphorylation and cyclin D1.

Finally, we investigated the mechanism of PIK3R3 over-expression in GC. Zhang *et al.* found PIK3R3 was over-expressed in ovarian cancer due to gene copy number gain [10]. However, no gene amplification or DNA de-methylation of PIK3R3 gene was found in our set of GC samples. Interestingly, Demoulin *et al.* found that PDGF activated transcription factor SREBP-1 in a PI3K-dependent manner [23], and ectopic expression of SREBP-1 induces the expression of PIK3R3 in AG01518 human foreskin fibroblasts [24]. Therefore, these results suggested that activated PI3K signaling could induce the expression of PIK3R3. Indeed, we observed a positive correlation between PIK3CA and PIK3R3 mRNA expression in our 126 GC samples. Furthermore, the PIK3CA inhibitor, LY294002 decreased PIK3R3 expression in HGC27 and IM95 GC cell lines. Our results thus provide direct experimental evidence that PIK3R3 expression can be regulated by PIK3CA.

## Conclusion

The *in silico* and molecular biological approaches used in the present study have provided a better understanding of the expression and role of PIK3R3 in GC. The identification of PIK3R3 as a proliferation-promoting factor in GC suggests that it might be a potential therapeutic target for a subset of GC.

## Abbreviations

PI3K: phosphatidylinositol 3-kinase; AML: acute myeloid leukaemia; GC: Gastric cancer; CBS: Circular Binary Segmentation; PI: propidium iodide; PBST: phosphate-buffered saline with 0.1% Tween 20; CGH: Comparative Genomic Hybridization; SH2: Src homology 2; N24: N-terminal 24 amino acid of PIK3R3.

## Competing interests

The authors declare that they have no competing interests.

## Authors’ contributions

JZ contributed to the lab work, interpreted the results and wrote the paper. GBC, YCT, and YW did statistical analyses of the data. RAS, CSY, GW, JH, and XX assisted the laboratory work. PT did the data collection. LKG designed the data analysis, interpreted the results and wrote the paper. PMY interpreted the results and wrote the paper. All authors have read and approved the final manuscript.

## Pre-publication history

The pre-publication history for this paper can be accessed here:

http://www.biomedcentral.com/1755-8794/5/34/prepub

## Supplementary Material

Additional file 1 ** Figure S1.** IPA pathway analysis of PIK3R3 co-regulated genes. Click here for file

Additional file 2 ** Figure S2.** Knock down PIK3R3 by siRNA (s16152, Invitrogen) inhibited the growth of HGC27 GC cells. (A) Western blot of PIK3R3 knockdown in transfected cells. (B) Cell number (measured by optical density (O.D.)) after knockdown by PIK3R3 siRNA. (C) DNA synthesis (BrdU incorporation) after knockdown of PIK3R3. The data in these experiments are expressed as means ± S.D. from three independent experiments. The significance was assessed by paired student’s *t*-test. *P < 0.05 comparing knockdown with control. (D) Cell death measured by Annexin-V/PI staining. These results using a different PIK3R3 siRNA gave similar results shown in Figure 2. Click here for file

Additional file 3 **Figure S3.** Effects of PIK3R3 knockdown on cell cycle progression and cell signaling of TMK1 GC cells. (A) The effect of PIK3R3 knockdown on cell cycle progression in TMK1 cells. The cells then were harvested 48 h after transfection followed by cell cycle analysis. The data are expressed as means ± S.D. from three independent experiments. The significance was assessed by paired student’s *t*-test. *P < 0.05 and **P < 0.01 compared with control. (B) Western blot analysis of the effects of PIK3R3 knockdown on cell signaling. Click here for file

Additional file 4 **Figure S4.** The correlation between the PIK3R3 mRNA expression (211580_s_at and 202743_at) and DNA copy number (A) or promoter methylation status (B) in 47 paired GC and control tissue. Click here for file

Additional file 5 **Figure S5.** The correlation between PIK3R3 and PIK3CA mRNA expression in 126 GC specimens. Click here for file
