# Supplementary figures and images for: Genetic and bioinformatic analyses of the expression and function of PI3K regulatory subunit PIK3R3 in an Asian patient gastric cancer library
Source: BMC Med Genomics. 2012 Aug 9;5:34. doi: 10.1186/1755-8794-5-34 (PMC3479415; doi:10.1186/1755-8794-5-34)

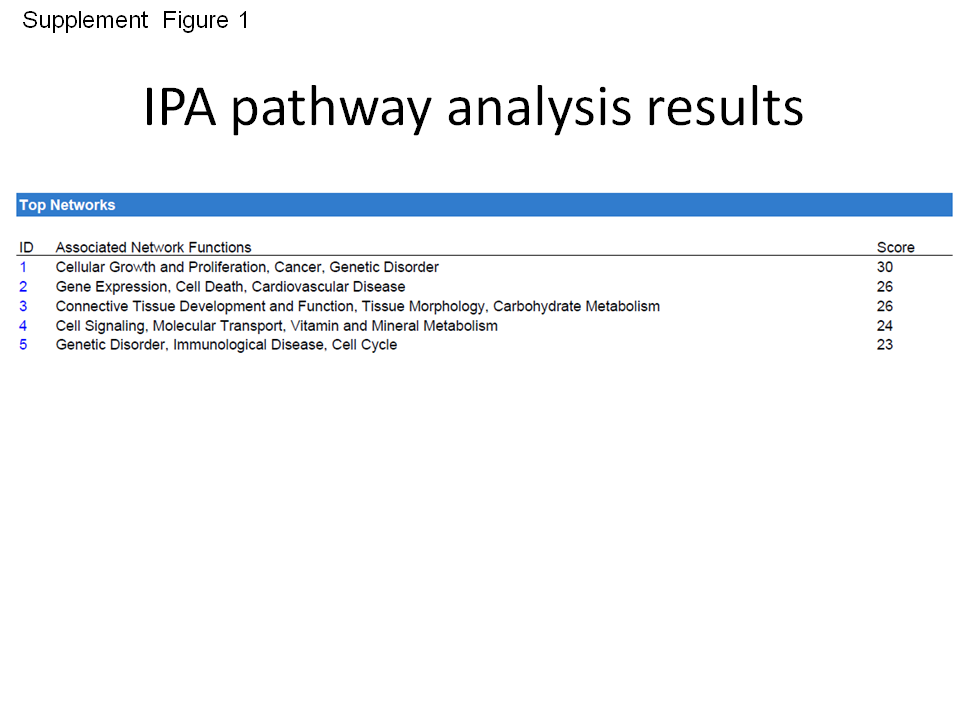

Supplement: Additional file 1 — Figure S1. IPA pathway analysis of PIK3R3 co-regulated genes. [file 1755-8794-5-34-S1.tiff]

## Slide 1
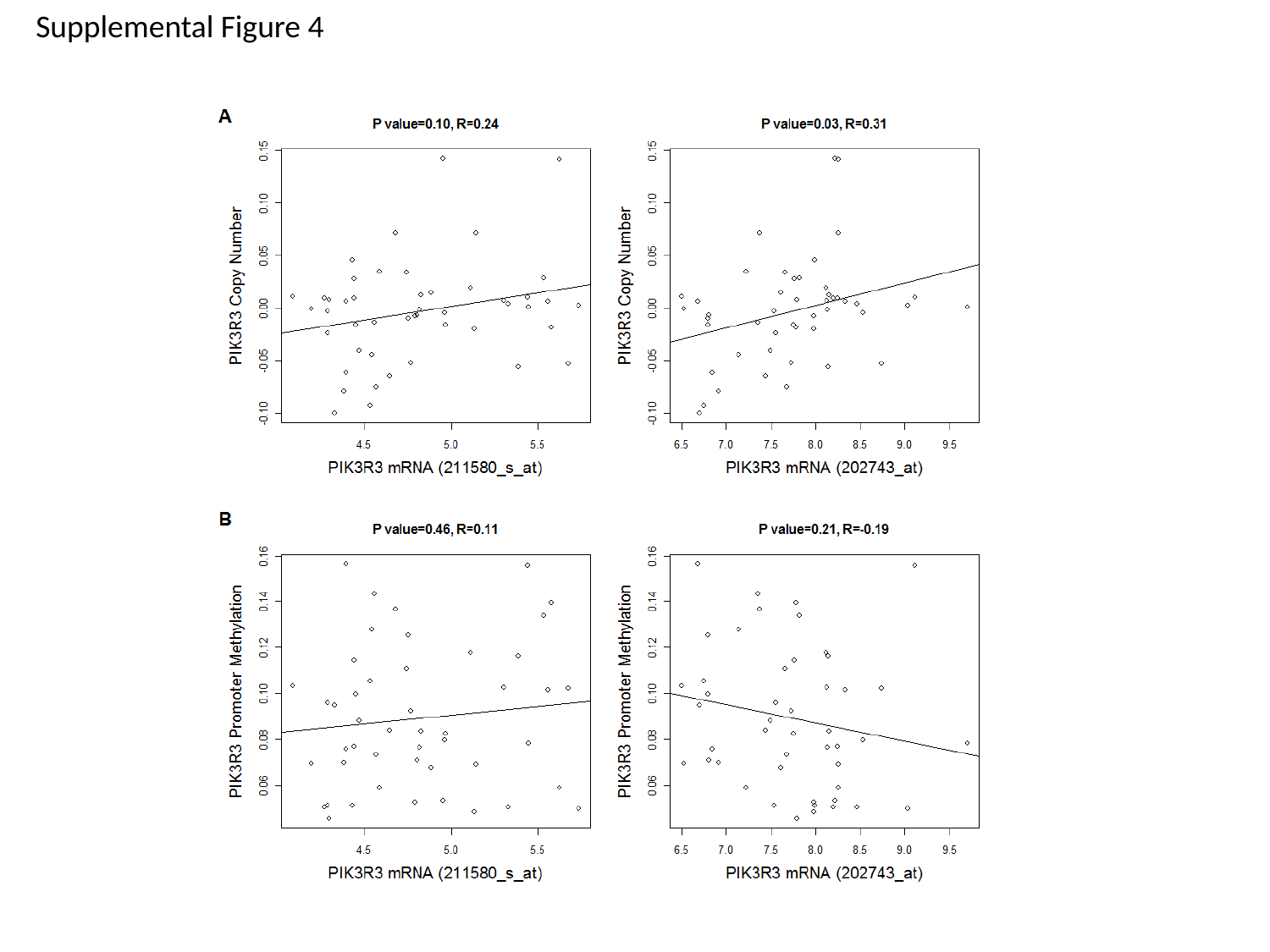

Supplemental Figure 4

Supplement: Additional file 4 — Figure S4. The correlation between the PIK3R3 mRNA expression (211580_s_at and 202743_at) and DNA copy number (A) or promoter methylation status (B) in 47 paired GC and control tissue. [file 1755-8794-5-34-S4.pptx]
