# Supplementary material for: Genetic and bioinformatic analyses of the expression and function of PI3K regulatory subunit PIK3R3 in an Asian patient gastric cancer library
Source: BMC Med Genomics. 2012 Aug 9;5:34. doi: 10.1186/1755-8794-5-34 (PMC3479415; doi:10.1186/1755-8794-5-34)
Supplement: Additional file 2 — Figure S2. Knock down PIK3R3 by siRNA (s16152, Invitrogen) inhibited the growth of HGC27 GC cells. (A) Western blot of PIK3R3 knockdown in transfected cells. (B) Cell number (measured by optical density (O.D.)) after knockdown by PIK3R3 siRNA. (C) DNA synthesis (BrdU incorporation) after knockdown of PIK3R3. The data in these experiments are expressed as means ± S.D. from three independent experiments. The significance was assessed by paired student’s t-test. *P < 0.05 comparing knockdown with control. (D) Cell death measured by Annexin-V/PI staining. These results using a different PIK3R3 siRNA gave similar results shown in Figure 2. [file 1755-8794-5-34-S2.pptx]

## Slide 1
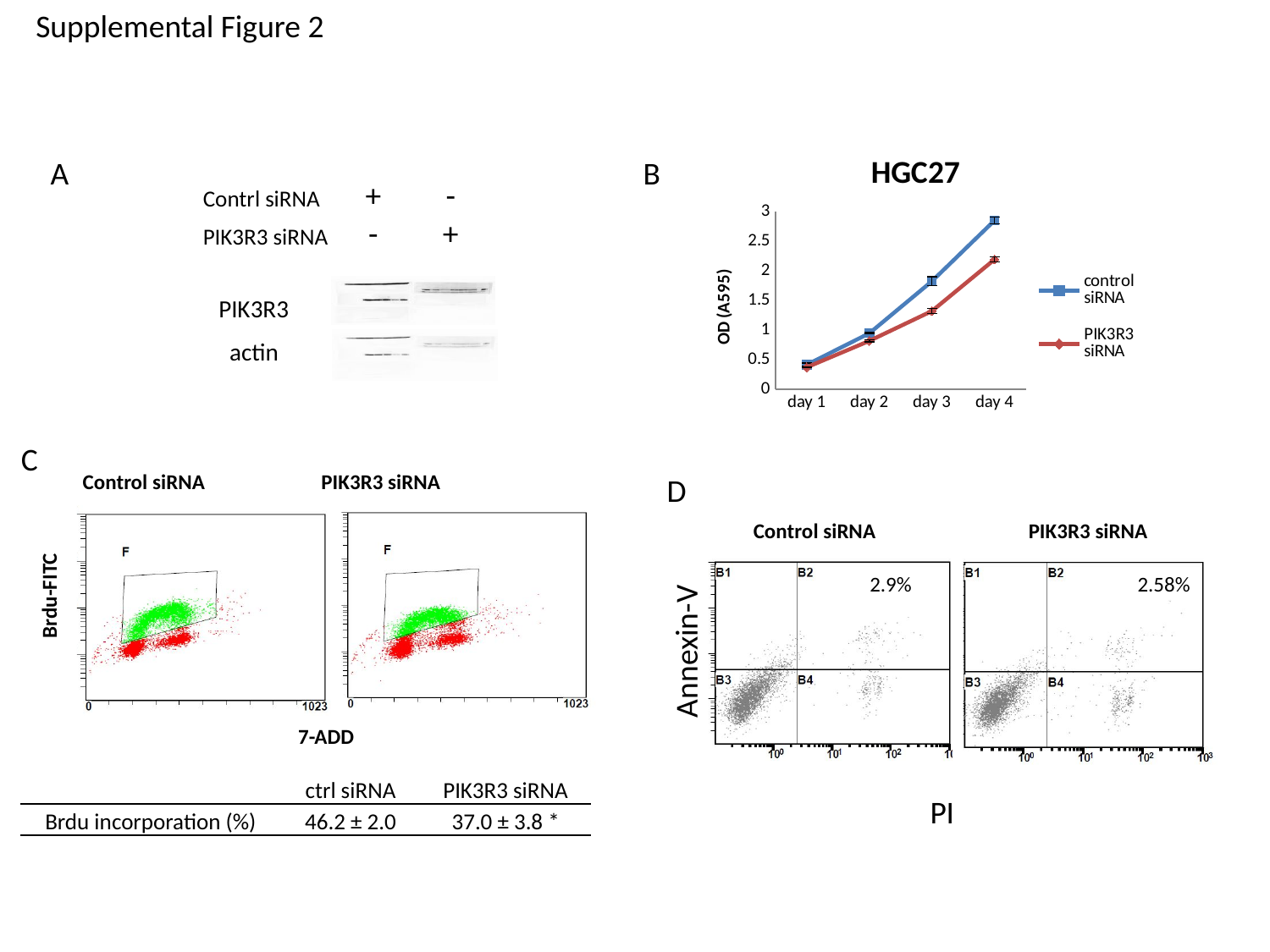

Supplemental Figure 2
### Chart: HGC27
| Category | control siRNA | PIK3R3 siRNA |
|---|---|---|
| day 1 | 0.4150000000000001 | 0.37266666666666676 |
| day 2 | 0.947 | 0.8180000000000002 |
| day 3 | 1.8256666666666668 | 1.322 |
| day 4 | 2.852 | 2.193666666666667 |A
B
Contrl siRNA + -
PIK3R3 siRNA - +
PIK3R3
actin
C
 Control siRNA PIK3R3 siRNA
D
Control siRNA
PIK3R3 siRNA
Brdu-FITC
Annexin-V
2.9%
2.58%
7-ADD
| | ctrl siRNA | PIK3R3 siRNA |
| --- | --- | --- |
| Brdu incorporation (%) | 46.2 ± 2.0 | 37.0 ± 3.8 \* |
PI
