# Supplementary material for: Genetic and bioinformatic analyses of the expression and function of PI3K regulatory subunit PIK3R3 in an Asian patient gastric cancer library
Source: BMC Med Genomics. 2012 Aug 9;5:34. doi: 10.1186/1755-8794-5-34 (PMC3479415; doi:10.1186/1755-8794-5-34)
Supplement: Additional file 3 — Figure S3. Effects of PIK3R3 knockdown on cell cycle progression and cell signaling of TMK1 GC cells. (A) The effect of PIK3R3 knockdown on cell cycle progression in TMK1 cells. The cells then were harvested 48 h after transfection followed by cell cycle analysis. The data are expressed as means ± S.D. from three independent experiments. The significance was assessed by paired student’s t-test. *P < 0.05 and **P < 0.01 compared with control. (B) Western blot analysis of the effects of PIK3R3 knockdown on cell signaling. [file 1755-8794-5-34-S3.pptx]

## Slide 1
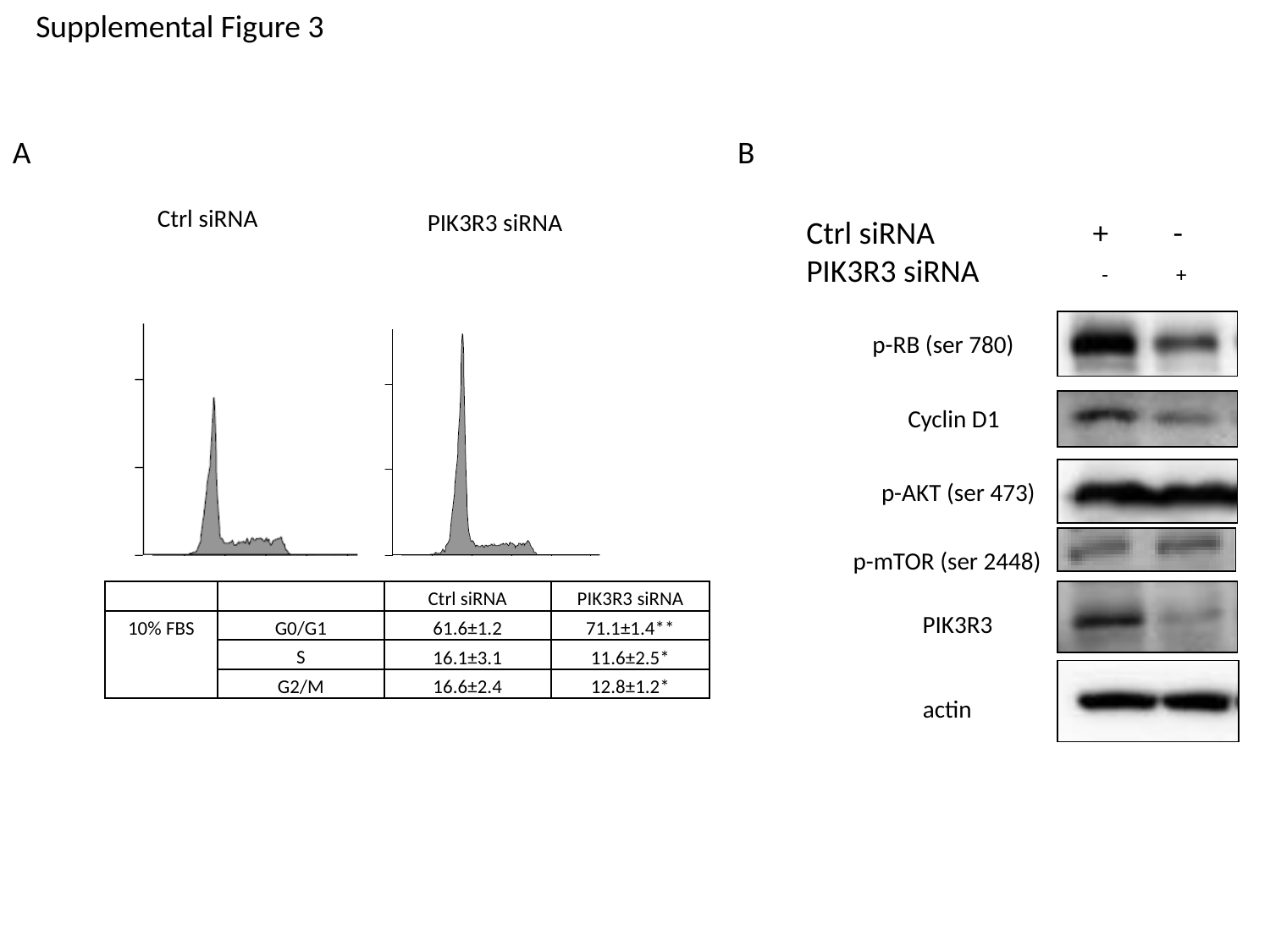

Supplemental Figure 3
A
B
Ctrl siRNA
PIK3R3 siRNA
Ctrl siRNA + -
PIK3R3 siRNA - +
p-RB (ser 780)
Cyclin D1
p-AKT (ser 473)
p-mTOR (ser 2448)
| | | Ctrl siRNA | PIK3R3 siRNA |
| --- | --- | --- | --- |
| 10% FBS | G0/G1 | 61.6±1.2 | 71.1±1.4\*\* |
| | S | 16.1±3.1 | 11.6±2.5\* |
| | G2/M | 16.6±2.4 | 12.8±1.2\* |
PIK3R3
actin
