# Supplementary material for: Genetic and bioinformatic analyses of the expression and function of PI3K regulatory subunit PIK3R3 in an Asian patient gastric cancer library
Source: BMC Med Genomics. 2012 Aug 9;5:34. doi: 10.1186/1755-8794-5-34 (PMC3479415; doi:10.1186/1755-8794-5-34)
Supplement: Additional file 5 — Figure S5. The correlation between PIK3R3 and PIK3CA mRNA expression in 126 GC specimens. [file 1755-8794-5-34-S5.pptx]

## Slide 1
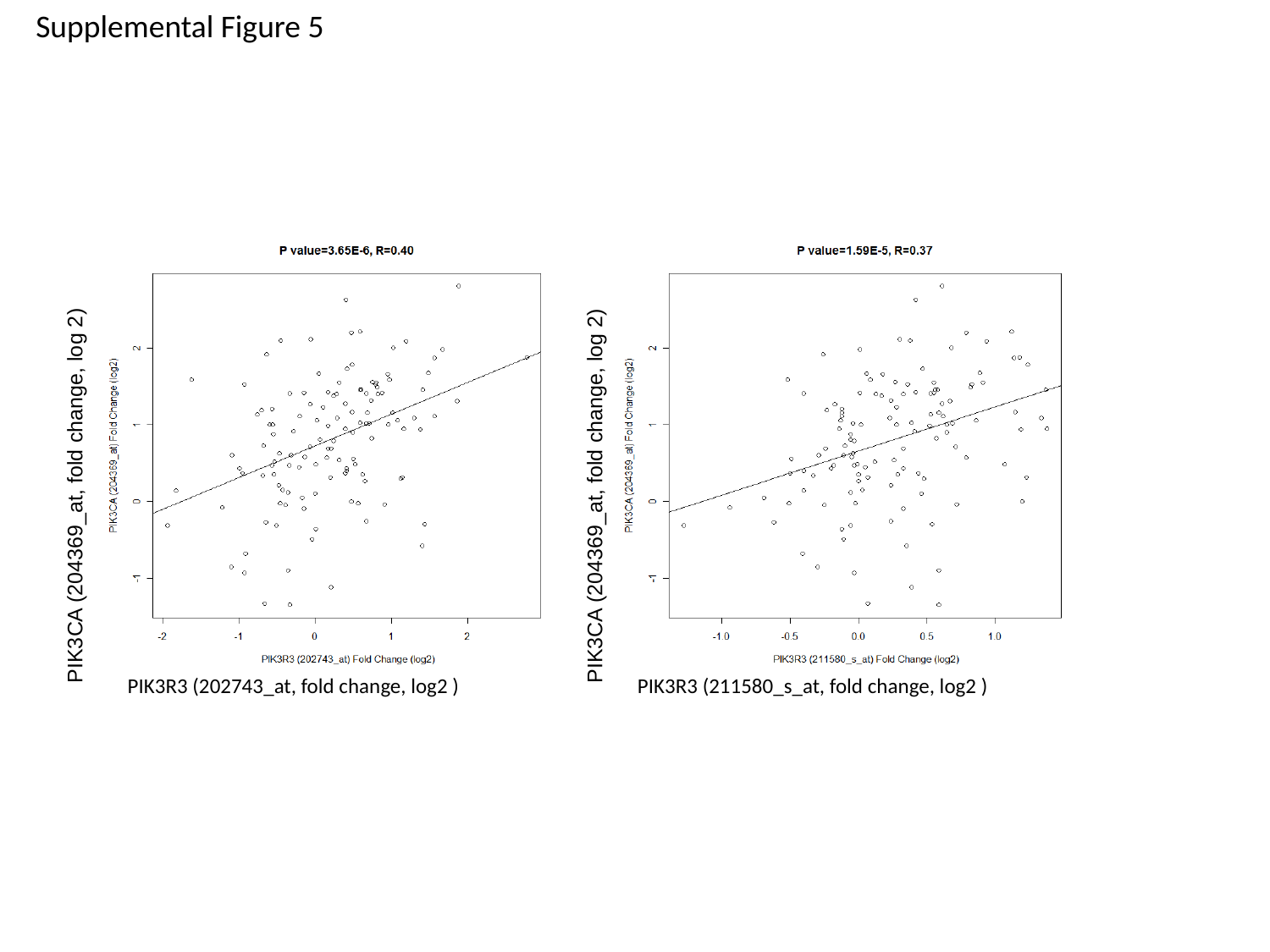

Supplemental Figure 5
PIK3CA (204369_at, fold change, log 2)
PIK3CA (204369_at, fold change, log 2)
PIK3R3 (202743_at, fold change, log2 )
PIK3R3 (211580_s_at, fold change, log2 )
